# Hemoglobin LjGlb1-1 is involved in nodulation and regulates the level of nitric oxide in the *Lotus japonicus–Mesorhizobium loti* symbiosis

**DOI:** 10.1093/jxb/erw290

**Published:** 2016-07-21

**Authors:** Mitsutaka Fukudome, Laura Calvo-Begueria, Tomohiro Kado, Ken-ichi Osuki, Maria Carmen Rubio, Ei-ichi Murakami, Maki Nagata, Ken-ichi Kucho, Niels Sandal, Jens Stougaard, Manuel Becana, Toshiki Uchiumi

**Affiliations:** ^1^Graduate School of Science and Engineering, Kagoshima University, 1-21-35 Korimoto, Kagoshima 890-0065, Japan; ^2^Departamento de Nutrición Vegetal, Estación Experimental de Aula Dei, Consejo Superior de Investigaciones Científicas, Apartado 13034, 50080 Zaragoza, Spain; ^3^Centre for Carbohydrate Recognition and Signalling, Department of Molecular Biology and Genetics, Aarhus University, Gustav Wieds Vej 10, 8000 Aarhus C, Denmark

**Keywords:** Hemoglobin, *Lotus japonicus*, *Mesorhizobium loti*, nitric oxide, nitrogen fixation, nodulation mutants, symbiosis.

## Abstract

Leghemoglobins transport and deliver O_2_ to the symbiosomes inside legume nodules and are essential for nitrogen fixation. However, the roles of other hemoglobins (Hbs) in the rhizobia–legume symbiosis are unclear. Several *Lotus japonicus* mutants affecting LjGlb1-1, a non-symbiotic class 1 Hb, have been used to study the function of this protein in symbiosis. Two TILLING alleles with single amino acid substitutions (A102V and E127K) and a *LORE1* null allele with a retrotransposon insertion in the 5′-untranslated region (96642) were selected for phenotyping nodulation. Plants of all three mutant lines showed a decrease in long infection threads and nodules, and an increase in incipient infection threads. About 4h after inoculation, the roots of mutant plants exhibited a greater transient accumulation of nitric oxide (NO) than did the wild-type roots; nevertheless, *in vitro* NO dioxygenase activities of the wild-type, A102V, and E127K proteins were similar, suggesting that the mutated proteins are not fully functional *in vivo*. The expression of LjGlb1-1, but not of the other class 1 Hb of *L. japonicus* (LjGlb1-2), was affected during infection of wild-type roots, further supporting a specific role for LjGlb1-1. In conclusion, the LjGlb1-1 mutants reveal that this protein is required during rhizobial infection and regulates NO levels.

## Highlight

Missense and null *Lotus japonicus* mutants allowed us to demonstrate that hemoglobin LjGlb1-1 is required for infection thread elongation and nodule formation, probably by regulating nitric oxide production in the roots.

## Introduction

Leghemoglobins are hemeproteins with a relatively high O_2_ affinity which are found exclusively in legume nodules (Appleby, 1984; Becana and Klucas, 1992; Smagghe *et al.*, 2009). They transport and deliver O_2_ to the symbiosomes at a steady but low concentration to avoid nitrogenase inactivation in bacteroids (Appleby, 1984) and are therefore essential for symbiotic N_2_ fixation (Ott *et al.*, 2005). However, hemoglobins (Hbs) are not confined to nodules but are ubiquitous in plant tissues, and can be categorized into classes 1, 2, and 3 according to their phylogenetic origin and biochemical properties (Trevaskis *et al.*, 1997; Watts *et al.*, 2001; Hunt *et al.*, 2002; Smagghe *et al.*, 2009).

Class 1 Hbs show extremely high O_2_ affinity that disqualifies them as O_2_ carriers or sensors (Smagghe *et al.*, 2009). In fact, several lines of evidence indicate that these Hbs are involved in the regulation of intracellular levels of nitric oxide (NO) (Igamberdiev and Hill, 2004; Hebelstrup *et al.*, 2013). This function may be ascribed in part to their NO dioxygenase (NOD) activity: NO+O_2_ **→**NO_3_^–^ (Igamberdiev and Hill, 2004; Perazzolli *et al.*, 2004). The expression of class 1 Hbs is induced in response to low temperature and hypoxia, conditions known to trigger NO production (Igamberdiev and Hill, 2004; Shimoda *et al.*, 2005). Treatment of plants with NO-releasing compounds, such as sodium nitroprusside (SNP) or *S*-nitroso-*N*-acetyl- d,l-penicillamine (SNAP), also induces expression of class 1 Hb genes (Shimoda *et al.*, 2005; Bustos-Sanmamed *et al.*, 2011). Overexpression of Arabidopsis class 1 Hb (AtGlb1) endows the plants with tolerance to hypoxic stress by decreasing the NO level (Hunt *et al.*, 2002), whereas silencing of the gene gives rise to stunted organs and delayed flowering (Hebelstrup and Jensen, 2008).

The roles of class 1 Hbs may be especially relevant during plant–microbe interactions. In plants, pathogens induce NO production, which in turn activates the expression of pathogenesis-related genes (Delledonne *et al.*, 1998; Durner *et al.*, 1998). NO is rapidly generated in both incompatible and compatible plant–pathogen combinations (Mur *et al.*, 2005), and also participates in disease resistance to necrotrophic pathogens (Asai and Yoshioka, 2009). Ectopic expression of *GhHb1*, a class 1 Hb gene of cotton (*Gossypium hirsutum*), increased the expression of the defense genes *PR1* and *PDF1.2* in Arabidopsis and enhanced resistance to the hemibiotrophic pathogen *Pseudomonas syringae* and the necrotrophic pathogen *Verticillium dahliae* (Qu *et al.*, 2006). On the other hand, an Arabidopsis line with silenced expression of AtGlb1 displayed increased resistance to *P. syringae* and *Botrytis cinerea* (Mur *et al.*, 2012). In this line, the levels of salicylate, jasmonate, and ethylene were increased in response to both pathogens. These findings suggest that class 1 Hbs are involved in the regulation of defense responses through NO and plant hormones. In some, but not all, combinations of symbiotic rhizobia and host plants, inoculation with rhizobia also induces NO generation in the host plant roots, concomitant with the expression of class 1 Hb genes (Nagata *et al.*, 2008). NO inhibits nitrogenase activity (Trinchant and Rigaud, 1982), but is also required for the onset of the *Medicago truncatula*–*Sinorhizobium meliloti* symbiosis (del Giudice *et al.*, 2011). Conversely, the addition of an NO scavenger enhanced the nitrogenase activity of nodules of *Alnus firma* and *Lotus japonicus* (Sasakura *et al.*, 2006; Shimoda *et al.*, 2009). Previous work by Uchiumi and colleagues has shown that overexpression of *LjGlb1-1* (Lj3g3v3338170; www.kazusa.or.jp/lotus/), a class 1 Hb gene of *L. japonicus*, results in a decrease of NO in nodules, with concomitant increases in nodulation and N_2_ fixation of the transformed hairy roots (Shimoda *et al.*, 2009). However, a conclusive proof of the involvement of class 1 Hbs in nodulation is lacking and requires the use of specific mutants because legumes express in nodules several Hbs of all three classes, in addition to leghemoglobins (Uchiumi *et al.*, 2002; Vieweg *et al.*, 2005; Bustos-Sanmamed *et al.*, 2011). In particular, the *L. japonicus* genome contains a second functional class 1 Hb gene, termed *LjGlb1-2* (Lj3g3v3338180; www.kazusa.or.jp/lotus/) (Bustos-Sanmamed *et al.*, 2011).

Here, the symbiotic phenotypes of *L. japonicus* mutant plants that are specifically affected in *LjGlb1-1* expression are reported. This was considered most relevant in the context of NO regulation in the symbiosis because *LjGlb1-1* is the only NO-inducible Hb gene of *L. japonicus* (Bustos-Sanmamed *et al.*, 2011). Two types of mutants, produced by TILLING (Perry *et al.*, 2009) and insertion of retrotransposon *LORE1* (Fukai *et al.*, 2012; Urbański *et al.*, 2012; Małolepszy *et al.*, 2016), were used in this work and shown to have lower infection rates, fewer nodules, and a higher NO level in roots than the wild-type (WT) plants. We conclude that LjGlb1-1 supports *M. loti* infection, probably by regulating the NO level in the roots of *L. japonicus.*

## Materials and methods

### Biological material and plant growth

Plants of WT and mutant lines of *L. japonicus* accession Gifu B-129 were used in this study. Seeds were gently scarified, disinfected with 2% sodium hypochlorite, washed, and left overnight in darkness. After imbibition, seeds were washed, left on plates with 0.5% agar for 3 d at 4 °C, transferred to agar plates containing nutritive medium, and placed vertically at 24 °C for 3 d in the dark. For phenotype analyses, seedlings were grown on Fåhraeus (1.5% agar) medium (Fåhraeus, 1957) and each seedling was inoculated with 10^6^ cells of *M. loti* strains MAFF303099 or MAFF303099 *Ds*Red (Maekawa *et al.*, 2009). For quantitative real-time PCR (qPCR) analyses, seedlings were grown on Jensen (0.9% agar) medium (Pajuelo and Stougaard, 2005) and inoculated with 10^7^ cells of *M. loti* strain R7A. Roots were harvested 1, 2, 4, and 6 d after inoculation, and were immediately flash-frozen in liquid nitrogen and stored at −80 °C until use. Uninoculated roots of the same age served as controls. In plates, roots were protected from light using black cardboard. For phenotyping non-nodulated plants, the Fåhraeus medium was supplemented with 1.5 mM NH_4_NO_3_. Plates were placed in growth cabinets with a 24 °C/21 °C day/night regime, 16h photoperiod, and 150 µmol m^−2^ s^−1^ light intensity.

### Mutant lines from TILLING and *LORE1* populations

TILLING was performed by RevGenUK (http://revgenuk.jic.ac.uk/), which provided M_3_ seeds carrying mutations in *LjGlb1-1*. Genomic DNA was isolated from leaves of M_4_ progeny with a DNeasy Plant Kit (Qiagen) according to the manufacturer’s instructions. Homozygous plants were selected by PCR with primers 5′-AGT CTA GAG TAA TCA CAT CAA TTC CAC C-3′ and 5′-TGA GTC TAA GAA GAT GAT GGC TTC A-3′ using a program consisting of 25 cycles at 94 °C for 30s, 57 °C for 1min, and 72 °C for 1min. The amplified products were sequenced. The M_5_ progeny derived from each homozygous individual were used for further experiments.

Null mutants bearing a retrotransposon insertion in the 5′-untranslated region (UTR) (line 30096642, hereafter abbreviated as 96642 to simplify) were obtained from the *LORE1* collection (Fukai *et al.*, 2012; Urbański *et al.*, 2012; Małolepszy *et al.*, 2016). The plants in the segregating population were genotyped by PCR using 96642-fw primer 5′-CAT GGC ATG AGG CTT GAG CTT GGG-3′ and 96642-rev primer 5′-TGA AAC CAC TCT CTT CTC GCC GCA-3′ to amplify the WT copy of *LjGlb1-1* and with 96642-fw primer and *LORE1* primer P2 5′-CCA TGG CGG TTC CGT GAA TCT TAG G-3′ to amplify the *LjGlb1-1* copy with the *LORE1* insertion. Three individual homozygous mutant plants were selected for analysis. Because their growth phenotypes did not significantly differ, the data obtained with the three individual mutants were pooled in this study. Both TILLING and *LORE1* mutant alleles originate from the *L. japonicus* Gifu ecotype (Handberg and Stougaard, 1992).

### Nodulation and nitrogenase activity

Plants grown as indicated earlier on Fåhraeus medium were inoculated with 10^6^ cells of *M. loti* MAFF303099 or its *Ds*Red-tagged derivative. Two weeks after inoculation, infection threads (ITs) were counted in two groups (incipient and long ITs) according to the terminology of Małolepszy *et al.* (2015), except that in our case the elongating ITs were also considered as long ITs. Four weeks after inoculation, nodules were detached and counted. The size and fresh weight of each plant were also measured. Nitrogenase activity of the detached nodules was determined as acetylene reduction activity according to Shimoda *et al.* (2009).

### Microscopic observation of endogenous and released NO in roots

Endogenous NO generation in roots of 5-day-old seedlings was monitored by fluorescence microscopy as described (Nagata *et al.*, 2008). Seedlings were inoculated with *M. loti* MAFF303099 or mock treated (sterile distilled water) and incubated for 3h. The seedlings were then soaked for 1h with 20 μM 4-amino-5-methylamino-2′,7′-difluorescein diacetate (DAF-FM DA; Sekisui Medical, Japan). In some experiments, the NO scavenger 2-(4-carboxyphenyl)-4,4,5,5-tetramethylimidazoline-1-oxyl-3-oxide (cPTIO) was applied at a concentration of 3 mM simultaneously with DAF-FM DA. Confocal images were captured with an A1si-90i microscope (Nikon, Japan) and epifluorescence images with an Eclipse 90i microscope (Nikon, Japan).

The concentration of NO released from the roots was assessed 4h after inoculation with *M. loti* MAFF303099. Roots were incubated with 7 μM DAF-FM for 3min, and the fluorescence intensity of the DAF-FM solution was measured as described (Tominaga *et al.*, 2009) with an ES-2 micro UV-visible fluorescence spectrophotometer (Malcom; Japan) using 495nm and 519nm as excitation and emission wavelengths, respectively. The released NO was expressed as relative fluorescence intensity per fresh weight of roots.

To investigate the effect of exogenous NO, 3-day-old seedlings were inoculated with *M. loti* MAFF303099 *Ds*Red, and 500 μM SNAP or 100 μM SNP was applied to the roots alone or with 500 μM cPTIO at the same time as inoculation. Two weeks after inoculation, the numbers of incipient and long ITs were counted.

### Production and mutagenesis of recombinant LjGlb1-1

The LjGlb1-1 protein (WT) was expressed in Champion pET200/D-TOPO vector (Invitrogen) as previously described in detail (Sainz *et al.*, 2013). The mutated derivative E127K was generated by PCR-based substitution by Mutagenex (Somerset, NJ, USA). Both the DNA and protein were sequenced to confirm the amino acid substitution. The proteins bearing an N-terminal poly-His tag were expressed in *Escherichia coli* C41 (DE3) cells (Lucigen) at 37 ºC for 4–6h with 0.2 mM isopropyl-β-d-1-thiogalactopyranoside. Cells were resuspended in 50 mM potassium phosphate (pH 7.5), broken by sonication, and cleared by centrifugation. The supernatant was fractionated with 30–75% ammonium sulfate, dialyzed in phosphate-buffered saline [PBS; 50 mM potassium phosphate (pH 7.5)+150 mM NaCl], and loaded on an Ni affinity column (HiTrap Chelating HP; GE Healthcare). The column was washed with 5 vols of PBS+20 mM imidazole, and the recombinant proteins were eluted with PBS+250 mM imidazole. The proteins were oxidized with ferricyanide, dialyzed in PBS, concentrated, quantified based on the Soret absorption band, and stored at −80 °C until analysis.

### Spectra and NOD activities of recombinant LjGlb1-1 proteins

The Soret–visible spectra of the LjGlb1-1 proteins (WT and E127K) in the ferric (Hb^3+^), deoxyferrous (Hb^2+^), and oxyferrous (Hb^2+^O_2_) states were recorded with a UV-visible Lambda 25 spectrophotometer (Perkin-Elmer). Hb^2+^ was produced by adding a trace of dithionite to Hb^3+^, and Hb^2+^O_2_ was formed by passing Hb^2+^ through a NAP-5 mini-column (GE Healthcare).

The NOD activities of the recombinant proteins were measured by using two NO donors and an NO-specific electrode (ISO-NOP), which was calibrated daily following the manufacturer’s instructions (World Precision Instruments, Sarasota, FL, USA). Diethylamine NONOate (DEA; 20 μM) and *S*-nitrosoglutathione (GSNO; 1mM) were added to 4ml of 50 mM potassium phosphate buffer (pH 7.5) containing 50 μM diethylenetriaminepentaacetic acid. This solution was kept with gentle shaking at 24 ºC until the NO concentration became stable (~4min), and freshly prepared Hb^2+^O_2_ protein (2 μM) was added and the decrease of NO measured. The time between the preparation of Hb^2+^O_2_ proteins and the NOD activity assay was always <5min. The Hb^3+^ proteins lacked NOD activity and were used as controls.

### Real-time quantitative PCR

Total RNA was extracted from 40–70mg of roots (6–10 roots) with the RNAqueous isolation kit (Ambion) and cDNA was synthesized using DNase-treated RNA with (dT)_17_ and Moloney murine leukemia virus reverse transcriptase (Promega). qPCR analysis was performed using a 7500 Real-Time PCR system (Applied Biosystems) and iTaq Universal SYBR Green Supermix reagents (Bio-Rad). Primers for *LjGlb1-1* (5′-TCT CAC TTC ACT TCC ATC GCA-3′ and 5′-TCA GTG AAA CAT GTG CTC CCA-3′) and *LjGlb1-2* (5′-GGC AGA AAA CAC AAC CAC CAT-3′ and 5′-TCA CCA CCA GAG CTT CTT GCT-3′) were used with a PCR program consisting of an intial denaturation and *Taq* polymerase activation step of 10min at 95 ºC, followed by 40 cycles of 15s at 95 ºC and 1min at 60 ºC, and a final melting curve stage. Primer specificity and the absence of contaminating genomic DNA were verified, respectively, by amplicon dissociation curves and by PCR analysis of RNA samples prior to reverse transcription. Expression levels were normalized using *ubiquitin* as reference gene, which was found to remain constant in roots during the few days of measurements.

## Results

### Growth and nodulation phenotypes of mutant LjGlb1-1 lines

In this work, two types of LjGlb1-1 mutants were used. On the one hand, mutant heterozygotes were identified by TILLING, each bearing a different amino acid substitution. Four mutant homozygous lines were established by self-pollination and designated as P59S, A102V, E127K, and E136K according to the amino acid substitutions and their positions. All of them showed a lower plant length than the WT, but only the A102V and E127K lines were selected for further studies because A102 is conserved and close to H104, which is important for assembling the heme moiety (Andersson *et al.*, 1996), and because E127 is highly conserved among class 1 plant Hbs (Supplementary Fig. S1 at *JXB* online). On the other hand, the mutant line 96642, which contains an insertion in the 5′-UTR of the *LjGlb1-1* gene 11bp upstream of ATG, was selected from the *LORE1* collection. Lines with annotated insertions in the coding parts of the exons of *LjGlb1-1* were not found in a total of 134 682 lines. Seedlings of the 96642 line were grown on agar plates and homozygous mutant plants were transferred to the greenhouse for seed production. Detailed qPCR analyses revealed that the *LjGlb1-1* mRNA level in roots of the A102V and E127K lines is similar to that of the WT, whereas it was virtually undetectable in the 96642 line (<1% of WT). This percentage was calculated considering the threshold value (*C*_t_) for *LjGlb1-1* in the 96642 (32–34 cycles) and WT (25–31 cycles) plants, together with the *C*_t_ value for *ubiquitin* (16–19 cycles). Consequently, the 96642 line can be considered as a null mutant.

**Fig. 1.**
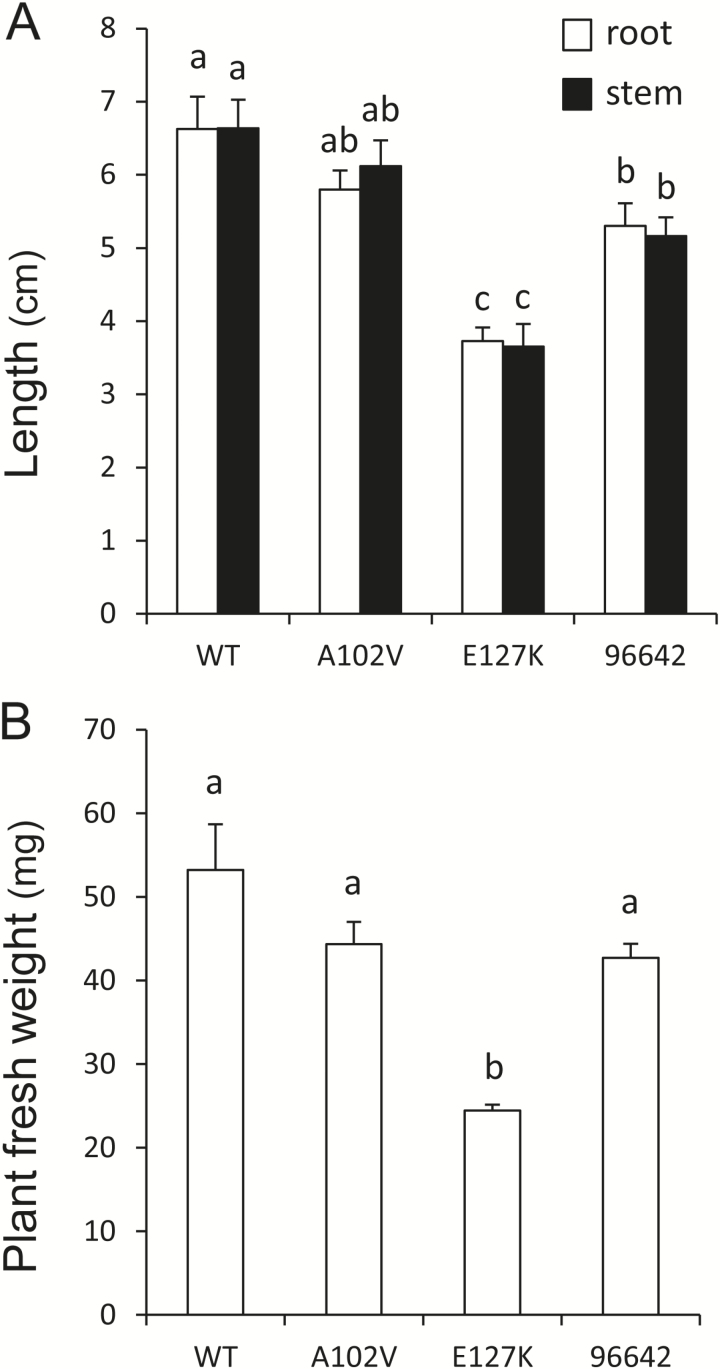
Growth phenotype of LjGlb1-1 mutant lines. Three days after germination, seedlings were inoculated with *M. loti* MAFF303099 and grown on nitrogen-free Fåhraeus medium for 4 weeks. At this time, (A) the root and stem lengths and (B) the fresh weight of plants were measured. Means (± SE; *n*=18 for WT, *n*=30 for 96642, *n*=12–16 for the other lines) denoted by the same letter do not significantly differ based on Duncan’s multiple range test at *P*=0.05.

**Fig. 2.**
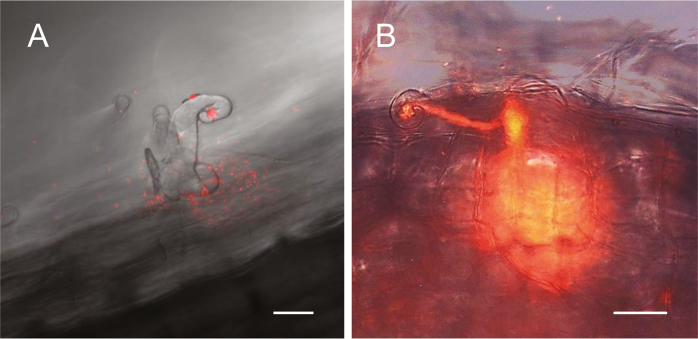
Typical (A) incipient IT and (B) long IT in roots of WT plants after 14 d of inoculation. Images were taken with a confocal microscope. Scale bars=25 μm.

The two TILLING and the single *LORE1* mutant lines were used for phenotyping, which included measurements of lengths and fresh weights of 4-week-old plants nodulated with *M. loti* MAFF303099. The E127K and 96642 plants had shorter roots and stems (Fig. 1A) and the E127K plants weighed less (Fig. 1B) than the WT. However, all the mutants maintained the same shoot length/root length ratio as the WT (Fig. 1A). The three mutant lines formed fewer nodules and the E127K and 96642 lines exhibited significantly lower N_2_ fixation activity, estimated as acetylene reduction activity per fresh weight of nodules (Table 1). The roots of the WT and mutant plants were inoculated with the *Ds*Red-labeled strain and the numbers of incipient ITs (microcolonies+short ITs) and long ITs (Fig. 2) were counted 2 weeks after inoculation. Compared with the WT plants, the numbers of incipient ITs were increased in the three mutant lines, whereas the numbers of long and total ITs were reduced (Table 1). Taken together, these observations indicate an alteration in the progression of ITs during infection in the roots of mutant plants.

**Table 1.** Nodulation phenotype of LjGlb1-1 mutant lines Seedlings were inoculated with *M. loti* MAFF303099 (nodule number and nitrogenase activity) or its *Ds*Red derivative (ITs) and were grown on nitrogen-free Fåhraeus medium. Nitrogenase (acetylene reduction) activity (ARA) of detached nodules was measured after 4 weeks and is expressed as nmol C_2_H_4_ produced min^−1^ mg^−1^ FW. The numbers of ITs were counted after 2 weeks and are expressed per centimeter of root.

	WT	A102V	E127K	96642
Nodule number	7.17±0.51 a	5.22±0.30 b	2.67±0.28 c	4.93±0.42 b
ARA	11.18±0.72 a	9.64±0.51 ab	2.71±0.60 c	8.54±0.69 b
Incipient ITs	2.88±0.13 a	4.43±0.42 b	8.15±1.28 b	4.52±0.61 b
Long ITs	32.08±1.41 a	17.11±0.98 b	5.98±0.93 c	11.57±0.82 d
Total ITs	34.95±1.42 a	21.54±1.30 b	14.00±2.05 c	16.09±1.21 c

Means (± SE, *n*=9 for ARA and *n*=14–22 for the other parameters) denoted by the same letter do not significantly differ (*P*=0.05) based on Duncan’s multiple range test.

Two additional phenotype measurements were carried out. First, the wt/wt siblings derived from M_4_ seeds of the A102V and E127K plants did not show significant differences in the plant length and in the nodule and IT numbers (Supplementary Table S1), which suggests that the mutations in the *LjGlb1-1* gene are responsible for the observed phenotypes. Secondly, because *LjGlb1-1* is expressed in roots and leaves in addition to nodules (Nagata *et al.*, 2008; Bustos-Sanmamed *et al.*, 2011), the phenotype was also analyzed in non-nodulated plants fed with NH_4_NO_3_ (Supplementary Table S2). Except for the shoot weight and length of the A102V plants, the growth parameters of the three mutant lines were decreased with respect to the WT. These results indicate that LjGlb1-1 does not only affect nodulation but is also involved in plant growth and development, as would be expected for a protein that is highly, but non-specifically, expressed in the nodules.

### Involvement of NO in the LjGlb1-1 nodulation phenotype

**Fig. 3.**
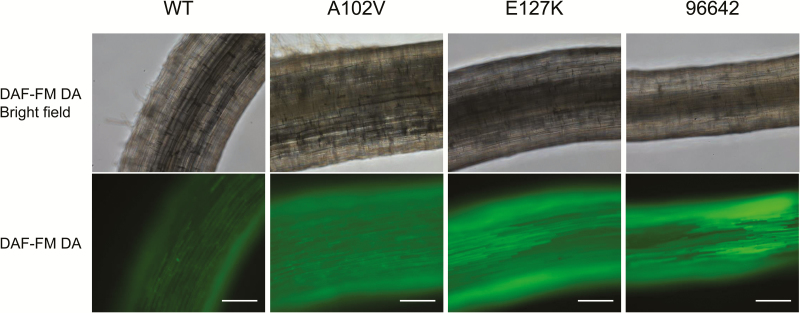
Fluorescence imaging of NO production in the roots of WT and mutant lines. Seedlings were incubated with *M. loti* MAFF303099 for 3h and then with DAF-FM DA for 1h. The images of the roots for all three lines were taken with a confocal microscope using identical settings. Roots incubated with mock (sterile distilled water) instead of rhizobia did not show detectable fluorescence. Scale bars=200 μm.

**Fig. 4.**
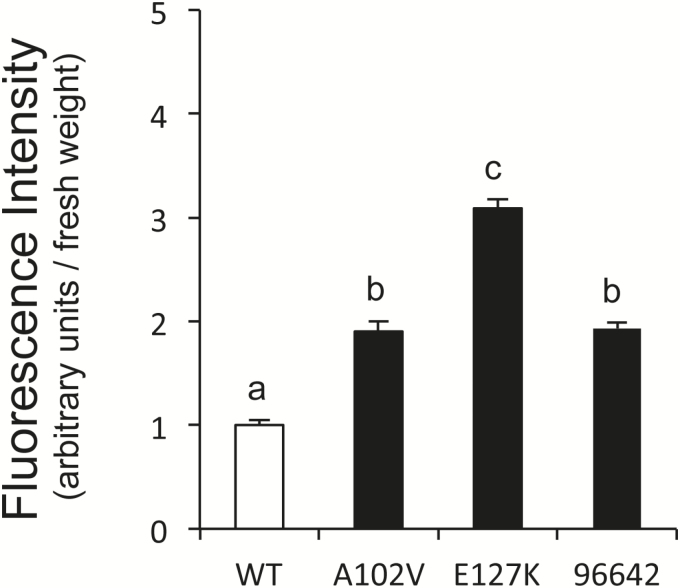
Quantification of NO released from roots of WT and mutant plants after incubation with *M. loti* MAFF303099. The fluorescence intensity of the DAF-FM solution in the rooting medium was measured with a plate reader and expressed as relative fluorescence intensity per fresh weight of roots. Means (± SE; *n*=9) denoted by the same letter do not significantly differ based on Duncan’s multiple range test at *P*=0.05.

The NO levels in the roots of WT and mutant plants were compared because class 1 Hbs may modulate the NO concentration *in vivo* (Hebelstrup *et al.*, 2013; Wally *et al.*, 2013). For this purpose, the NO-specific fluorescent dye DAF-FM DA was applied to the roots 3h after inoculation with *M. loti* and then incubated for 1h. This compound is cell permeant and essentially non-fluorescent, but it is deacetylated by intracellular esterases to DAF-FM, which in turn reacts with endogenous NO forming a highly fluorescent benzotriazole. In the WT roots, the fluorescence intensity, marking the production of NO, increased very slightly 4h after inoculation with *M. loti* (Fig. 3), consistent with a previous report (Nagata *et al.*, 2008). However, the signal intensity was enhanced in the A102V, E127K, and 96642 mutants compared with the WT (Fig. 3). This signal was abolished by incubation with the NO-specific scavenger cPTIO, thus confirming that NO was the reactive molecule being detected (Supplementary Fig. S2). Consequently, endogenous NO was low and increased slightly in the WT roots shortly after infection, but this transient increase was higher in the mutant roots. The NO released from roots was quantified by using the non-permeant dye DAF-FM and expressed as the relative fluorescence signal per fresh weight of roots. The fluorescence intensity was significantly higher in the roots of the three mutant plants than in the roots of WT plants (Fig. 4), in agreement with the observed increase of NO production by the roots of both mutants lines.

To investigate further whether NO was mediating, at least in part, the nodulation phenotype of the mutants, SNAP was applied to roots of WT plants at the same time as inoculation. Treatment with this NO donor resulted in an increase in the number of incipient ITs and in a reduction of the numbers of long and total ITs (Table 2). The addition of cPTIO alone or together with SNAP reverted completely or partially the effect of SNAP on those parameters, pointing to NO as the responsible reactive molecule (Table 2). Similar increasing (incipient ITs) or decreasing (long ITs) effects were observed upon the treatment of roots with SNP, another NO donor (Supplementary Table S3).

**Table 2.** Effect of NO on nodulation of WT plants Seedlings were inoculated with *M. loti* MAFF303099 *Ds*Red and 100 μl of 500 μM SNAP, 500 μM cPTIO, or a combination of both compounds, was applied on each root. The numbers of ITs are expressed per centimeter of root. All parameters were measured 2 weeks after inoculation.

	Control	SNAP	cPTIO	SNAP+cPTIO
Incipient ITs	2.7±0.3 a	8.0±1.1 b	4.9±0.6 b	3.8±0.5 a
Long ITs	25.4±2.7 a	9.8±1.2 b	28.7±2.4 a	26.0±3.3 a
Total ITs	28.1±3.0 a	17.8±1.9 b	33.6±2.4 a	29.7±3.7 a

Means (± SE, *n*=9–11) denoted by the same letter do not significantly differ (*P*=0.05) based on Duncan’s multiple range test.

### Characterization of mutated LjGlb1-1

**Fig. 5.**
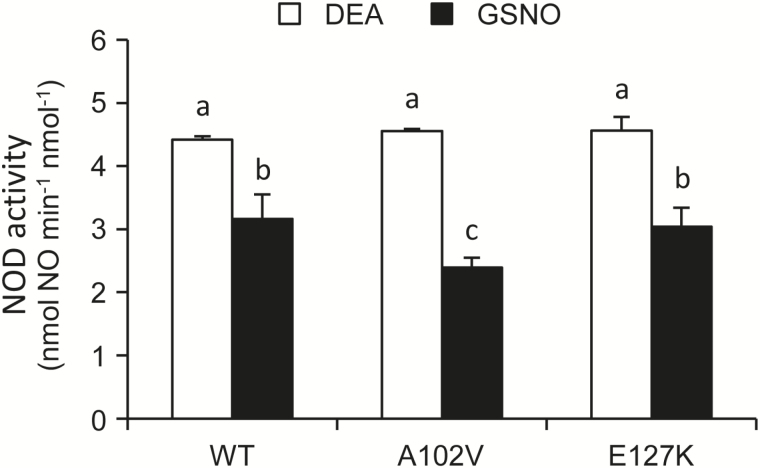
NOD activity of recombinant LjGlb1-1 WT and its A102V and E127K mutant derivatives. The activity was measured with 20 μM DEA or 1mM GSNO and 2 μM oxyferrous proteins in 50 mM potassium phosphate buffer (pH 7.5) containing 50 μM diethylenetriaminepentaacetic acid. Means (± SE; *n*=2–3 independent protein preparations) denoted by the same letter do not significantly differ based on Duncan’s multiple range test at *P*=0.05.

The possibility that the increased NO accumulation observed in roots of A102V and E127K plants was due to an alteration of the biochemical properties of the mutated proteins was explored. To this end, recombinant WT, A102V, and E127K proteins were purified by metal-affinity chromatography. The preparations had a purity of >90% and were free of contaminating *E. coli* flavohemoglobin (44 kDa) according to Coomassie-stained SDS gels (Supplementary Fig. S3). The absence of contaminating Hb from *E. coli* was further confirmed using the same vector and purification protocol from bacterial extracts for glutathione peroxidase, which lacked NOD activity as expected for a non-heme protein. The Soret–visible spectra (Supplementary Fig. S4) and NOD activities (Fig. 5) of the three proteins were compared. The spectra were similar, indicating that the hemes in the mutated proteins are hexacoordinated in the ferric and deoxyferrous forms (Smagghe *et al.*, 2009; Sainz *et al.*, 2013) and are still able to bind O_2_. This was confirmed by the ability of the A102V and E127K proteins to carry out the NOD reaction, which requires both O_2_ and NO binding inside the heme cavity. In fact, the mutated proteins displayed *in vitro* similar NOD activities to the WT protein using DEA or GSNO as NO donors (Fig. 5). Consequently, the potential of the mutant proteins to scavenge NO through its dioxygenation to NO_3_^−^ remains intact.

### Expression profiles of LjGlb1-1 and LjGlb1-2 during infection

**Fig. 6.**
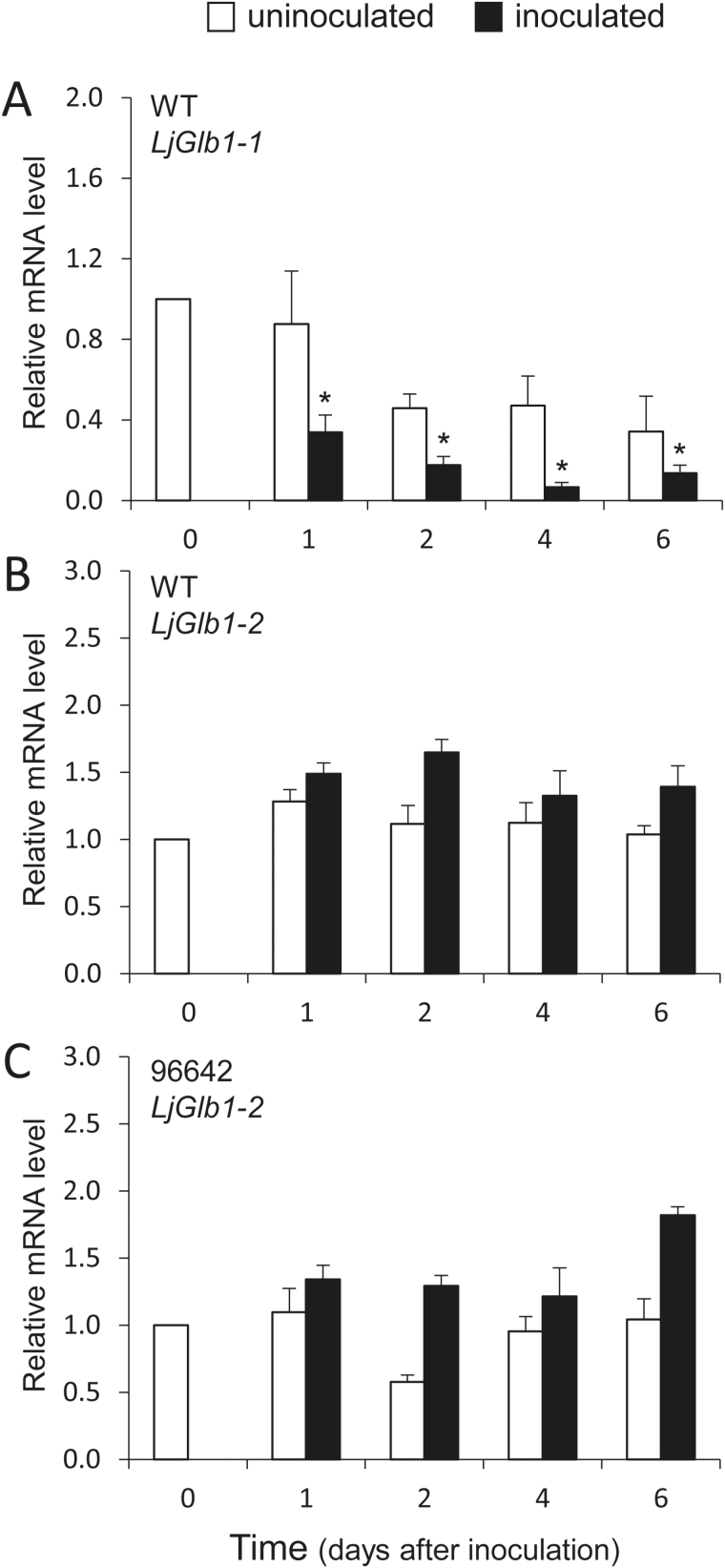
Expression analysis of the *LjGlb1-1* and *LjGlb1-2* genes in roots during the first days upon inoculation with *M. loti* R7A. Transcript steady-state levels were expressed relative to the day of inoculation (day 0). The figure shows expression levels of *LjGlb1-1* (A) and *LjGlb1-2* (B) in roots of WT plants, and of *LjGlb1-2* (C) in roots of 96642 plants. Values are means ± SE of four biological replicates (different RNA extractions), each with three technical replicates. White and black bars represent values for uninoculated and inoculated roots, respectively. The asterisk denotes significant gene down-regulation (relative mRNA level <0.5).

The *L. japonicus* genome encodes two class 1 Hbs, but only LjGlb1-1 appears to be induced by NO at the transcriptional level (Shimoda *et al.*, 2005; Bustos-Sanmamed *et al.*, 2011). It was therefore of interest to determine by qPCR the expression profiles of the *LjGlb1-1* and *LjGlb1-2* genes in roots of WT plants during the first days of infection (Fig. 6). The expression profile of *LjGlb1-2* was also obtained for roots of the 96642 line, which shows undetectable *LjGlb1-1* mRNA levels. In these experiments, uninoculated roots of the same age were used for comparison (Fig. 6, white bars). The *LjGlb1-1* mRNA level of inoculated roots decreased between 1d and 6 d after infection (Fig. 6A, black bars), whereas the *LjGlb1-2* mRNA level was not affected (Fig. 6B, black bars). The transcript levels of both genes remained constant in the uninoculated root controls (Fig. 6A, B, white bars). No changes were observed for the *LjGLb1-2* mRNA levels in the roots of the 96642 mutant (Fig. 6C, white and black bars).

## Discussion

Previous work in one of our laboratories using transgenic hairy roots of *L. japonicus* has shown that overexpression of *LjGlb1-1* improves nodule formation, decreases the NO level, and enhances nitrogenase activity of nodules (Shimoda *et al.*, 2009). These observations led us to surmise that a deficiency of LjGlb1-1 in plants would reduce nodulation and nitrogenase activity. To test this hypothesis, three mutant lines of *L. japonicus* were used in this study. The A102V and E127K lines express proteins bearing single mutations in conserved amino acid residues, whereas the 96642 line is unable to express the protein. The E127K and 96642 plants showed shorter roots and stems and the E127K plants had lower fresh weight (Fig. 1). The three mutants were also altered in nodulation, with decreases of the nodule number, long ITs, and acetylene reduction activity, and an increase of incipient ITs (Table 1). However, the plant growth and nodulation were more inhibited in E127K than in A102V and 96642, whereas w/w siblings of both A102V and E127K plants did not show this phenotype (Supplementary Table S1). A similar more intense phenotype was observed for E127K plants grown under non-nodulating conditions, which also disppears in the w/w siblings (Supplementary Table S2). Thus, it may be that the E127K line carries an additional linked mutation causing, at least in part, a more penetrating phenotype. The reduction in the number of long ITs in the roots of the three mutant lines indicates that the infection process is restricted at the initial stages of rhizobial entry, particularly during progression of ITs. This alteration may, in turn, increase in the number of attempted infections, as observed for other mutants (Wopereis *et al.*, 2000).

The results of this work also reveal that this negative effect on nodulation is due, at least in part, to deregulation of NO levels in the roots. Thus, NO accumulates in roots after only ~4h from infection, confirming our previous observation (Shimoda *et al.*, 2005). Here, the accumulation of NO was found to be enhanced in the roots of the three mutant lines, providing strong support for our hypothesis that LjGlb1-1 regulates endogenous NO levels during infection so as to avoid triggering of a defense response in roots (Shimoda *et al.*, 2005). The transient increase of endogenous NO would therefore explain the alteration of the infection process, as evidenced by the changes in the nodule and IT numbers in the mutant plants (Table 1). This proposal is further supported by our observation that exogenous NO, provided by SNAP or SNP, also inhibits IT elongation (Table 2) The reason why the roots of the A102V and E127K plants accumulate higher NO levels than those of the WT plants is nevertheless uncertain because the two mutated proteins and the WT protein exhibit similar NOD activities *in vitro* (Fig. 5). Two plausible and compatible explanations are that the NOD activity is being limited *in vivo* by a reductant that is essential to regenerate the ferrous globin (Becana and Klucas, 1992; Igamberdiev and Hill, 2004; Smagghe *et al.*, 2008; Sainz *et al.*, 2013), and that the mutations affect protein stability in the intracellular medium. An alternative explanation that cannot be discarded, but seems more unlikely, is that the higher accumulation of NO in the mutants is indirectly originated by LjGlb1-1 malfunction rather than by a decreased capacity for NO scavenging.

The expression analysis of the two class 1 Hb genes of *L. japonicus* shows that *LjGlb1-1* mRNA levels decrease within the first days of infection (Fig. 6A). Previous work has shown that *LjGlb1-1* is induced by NO after only 3–4h (Shimoda *et al.*, 2005; Bustos-Sanmamed *et al.*, 2011), and therefore NO and *LjGlb1-1* expression levels might change in parallel during infection. In this scenario, NO accumulation and hence *LjGlb1-1* induction are restricted to a short time frame, and afterwards, between 1 d and 6 d, both NO and *LjGlb1-1* mRNA levels decline (Shimoda *et al.*, 2005; Fig. 6A). In sharp contrast, *LjGlb1-2* expression remained unaffected (Fig 6B, C), which allows us to conclude that this gene is not involved in the NO-dependent response of the roots to rhizobial infection.

In the *M. truncatula–S. meliloti* interaction, NO is required for the optimal establishment of the symbiosis, and a decrease of NO production in the roots inhibits nodulation (del Giudice *et al.*, 2011; Pauly *et al.*, 2011). Our results, including those of Shimoda *et al.* (2005) and Nagata *et al.* (2008), appear to contradict these reports, and so far there is no clear explanation for this discrepancy, which may be ascribed to differences between the indeterminate (*M. truncatula*) and determinate (*L. japonicus*) patterns of nodule development and/or in the range of NO concentrations needed for the onset of the two symbioses. In any case, the use of LjGlb1-1 mutants in the present study provides conclusive evidence that this particular class 1 Hb is essential for proper nodulation as its deficiency results in a reduced number of elongated ITs caused, at least in part, by the transient accumulation of NO in the infected roots.

## Supplementary data

Supplementary data are available at *JXB* online.

Table S1. Growth and nodulation phenotype of WT plants and w/w sibblings (M_5_ seeds) from mutant plants.

Table S2. Growth parameters of non-nodulated LjGlb1-1 mutant plants, and derived w/w siblings, supplied with combined nitrogen.

Table S3. Effect of SNP application to roots on nodulation of WT plants.

Figure S1. Alignment of some class 1 Hbs showing conservation of A102 and E127.

Figure S2. Inhibition of NO-associated fluorescence by cPTIO in roots of WT and mutant plants.

Figure S3. Purification of WT recombinant LjGlb1-1.

Figure S4. Representative Soret–visible spectra of recombinant WT, A102V, and E127K proteins.

Supplementary Data

## Abbreviations:

Hb: hemoglobin
IT: infection thread
NO: nitric oxide
NOD: nitric oxide dioxygenase
SNAP: *S*-nitroso-*N*-acetyl-d,l-penicillamine
SNP: sodium nitroprusside.
