# Supplementary material for: Hemoglobin LjGlb1-1 is involved in nodulation and regulates the level of nitric oxide in the Lotus japonicus–Mesorhizobium loti symbiosis
Source: J Exp Bot. 2016 Jul 21;67(17):5275–83. doi: 10.1093/jxb/erw290 (PMC5014168; doi:10.1093/jxb/erw290)
Supplement: Supplementary Data [file supp_67_17_5275__index.html]

Hemoglobin LjGlb1-1 is involved in nodulation and regulates the level of nitric oxide in the Lotus japonicus–Mesorhizobium loti symbiosis — Hemoglobin LjGlb1-1 is involved in nodulation and regulates the level of nitric oxide in the Lotus japonicus–Mesorhizobium loti symbiosis — Supplementary Data 

# Hemoglobin LjGlb1-1 is involved in nodulation and regulates the level of nitric oxide in the *Lotus japonicus–Mesorhizobium loti* symbiosis

## Supplementary Data

Data files

- supplementary\_tables\_S1\_S3\_figures\_S1\_S4.pdf - Supplementary Data
