# Supplementary material for: Hemoglobin LjGlb1-1 is involved in nodulation and regulates the level of nitric oxide in the Lotus japonicus–Mesorhizobium loti symbiosis
Source: J Exp Bot. 2016 Jul 21;67(17):5275–83. doi: 10.1093/jxb/erw290 (PMC5014168; doi:10.1093/jxb/erw290)
Supplement: Supplementary Data [file supp_erw290_supplementary_tables_S1_S3_figures_S1_S4.pdf]

Table S1. *Growth and nodulation phenotype of WT plants and wild-type homozygous sibblings (M5 seeds) from mutant plants*

Plant length is expressed in centimeters and the number of ITs per centimeter of root. Values are means ± SE (*n*= 60 for plant length and nodule number; *n*=10-20 for ITs). Means denoted by the same letter do not significantly differ (*P*=0.05) based on the Duncan's multiple range test.

	**WT**	**A102V w/w**	**E127K w/w**
Plant length	14.3 ± 0.5 a	12.8 ± 0.8 a	13.5 ± 0.4 a
Nodule number	6.8 ± 0.2 a	6.6 ± 0.2 a	6.3 ± 0.3 a
Incipient ITs	2.9 ± 0.1 a	2.5 ± 0.2 a	2.5 ± 0.2 a
Long ITs	32.0 ± 1.4 a	28.4 ± 1.0 a	28.8 ± 1.3 a
Total ITs	35.0 ± 1.4 a	30.9 ± 1.1 a	31.3 ± 1.4 a

Table S2. *Growth parameters of non-nodulated LjGlb1-1 mutant plants, and derived wild-type homozygous siblings, supplied with combined nitrogen*

Plants were grown on Fåhraeus medium supplemented with 1.5 mM NH_4NO_3 for three weeks. Lengths are expressed in centimeters and weights in grams. Means (± SE, *n*= 9-12) denoted by the same letter do not significantly differ (*P*=0.05) based on the Duncan's multiple range test.

	**WT**	**A102V**	**E127K**	**96642**	**A102V (w/w)**	**E127K (w/w)**
Shoot length	4.68 ± 0.09 a	4.33 ± 0.20 ab	3.29 ± 0.24 c	3.86 ± 0.15 b	4.69 ± 0.17 a	4.56 ± 0.12 a
Shoot weight	29.83 ± 1.19 a	27.00 ± 1.65 a	20.67 ± 1.73 b	26.31 ± 0.87 a	30.11± 1.67 a	29.89 ± 1.11 a
Root length	5.37 ± 0.21 a	4.13 ± 0.23 b	2.00 ± 0.33 c	3.73 ± 0.22 b	5.40 ± 0.18 a	5.09 ± 0.14 a
Root weight	29.25 ± 1.04 a	23.89 ± 2.21 b	12.67 ± 0.87 c	24.54 ± 1.53 b	29.78 ± 1.19 a	27.56 ± 1.31 a
Leaf number	4.92 ± 0.15 a	4.11 ± 0.31 b	2.56 ± 0.45 c	3.92 ± 0.14 b	4.89 ± 0.11 a	4.78 ± 0.15 a

Table S3. *Effect of SNP application to roots on nodulation of WT plants*

Seedlings were inoculated with *M. loti* MAFF303099 DsRed and grown on nitrogen-free Fåhraeus medium for four weeks. The numbers of ITs are expressed per centimeter of root. Means (± SE, *n*= 9-11) denoted by different letters significantly differ (*P*=0.05) based on the Student's *t*-test.

	**Control**	**SNP**
Incipient ITs	3.6 ± 0.4 a	10.5 ± 0.7 b
Long ITs	25.5 ± 2.1 a	6.6 ± 0.7 b
Total ITs	29.1 ± 2.1 a	17.2 ± 1.2 b

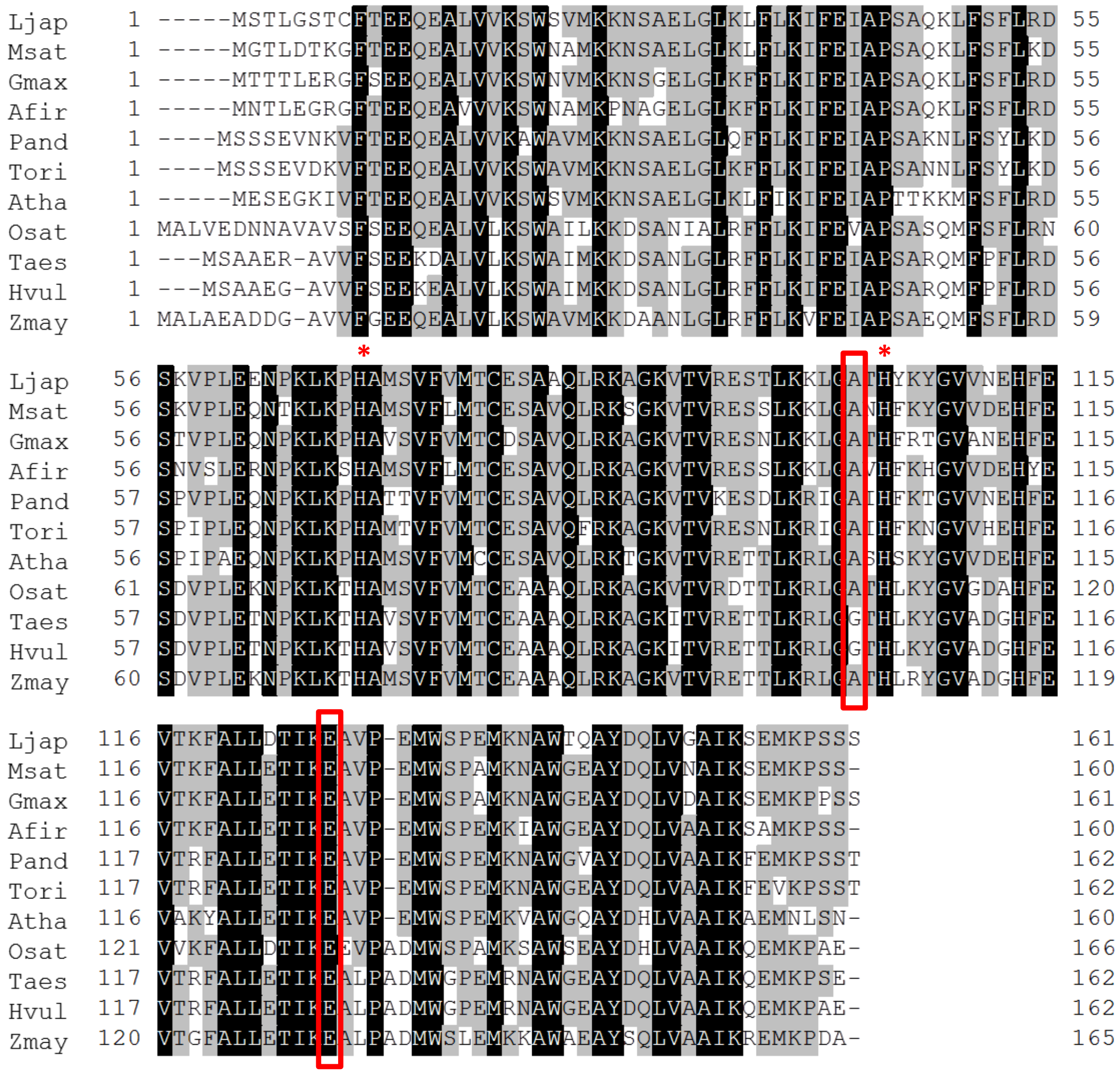

Figure S1: Alignment of some class 1 Hbs showing conservation of A102 and E127. Both residues are within red boxes and histidine residues that are important for heme assembly are marked with red asterisks. *Abbreviations* (accession numbers in parentheses): Afir, *Alnus firma* (BAE75956); Atha, *Arabidopsis thaliana* (AEC06463); Gmax, *Glycine max* (AAA97887); Hvul, *Hordeum vulgare* (AAB70097); Ljap, *Lotus japonicus* (BAE46739); Msat, *Medicago sativa* (AAG29748); Osat, *Oryza sativa* (AAM19125); Pand, *Parasponia andersonii* (AAB86653); Taes, *Triticum aestivum* (AAN85432); Tori, *Trema orientalis* (AAC28426); Zmay, *Zea mays* (AAG01375).

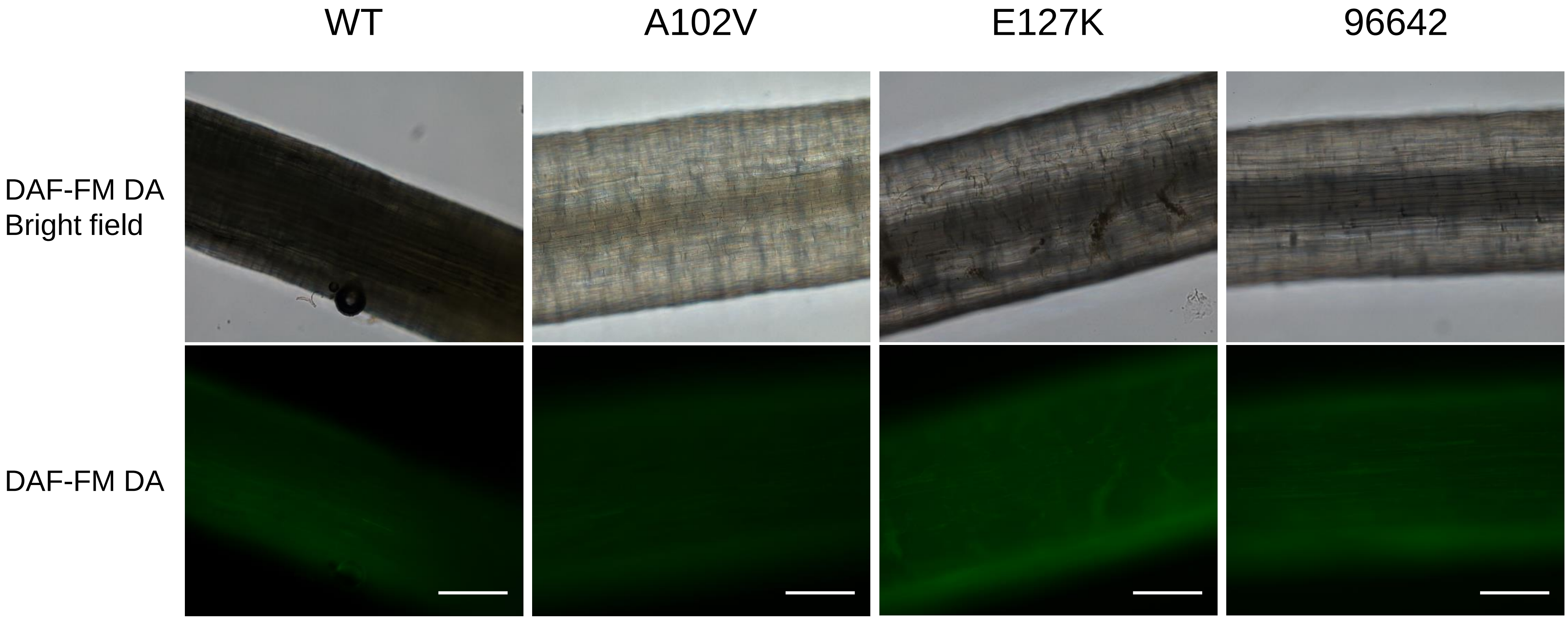

Figure S2. Inhibition of NO-associated fluorescence by cPTIO in roots of WT and mutant plants. The figure shows representative epifluorescence and bright-field images of roots after 1-h incubation with 20 μM DAF-FM DA combined or not with 3 mM cPTIO. Bars, 200 μm.

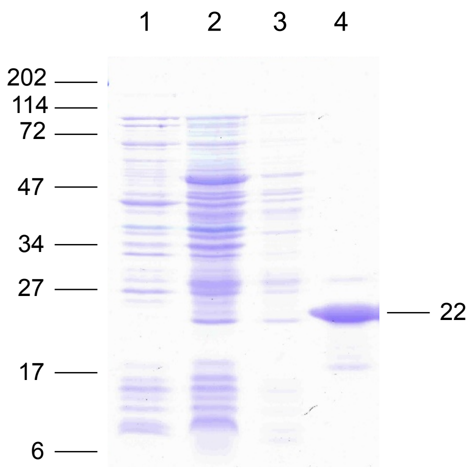

Figure S3: Purification of wild-type recombinant LjGlb1-1. SDS-gels (12.5%) stained with Coomassie blue. Lanes (5 μg protein): 1, preinduced culture; 2, induced with 0.25 mM IPTG; 3, after ammonium sulfate (30-75%) fractionation; 4, after Ni-affinity chromatography. Molecular mass markers (kDa) are shown on the left. Similar results were obtained for the mutated versions A102V and E127K.

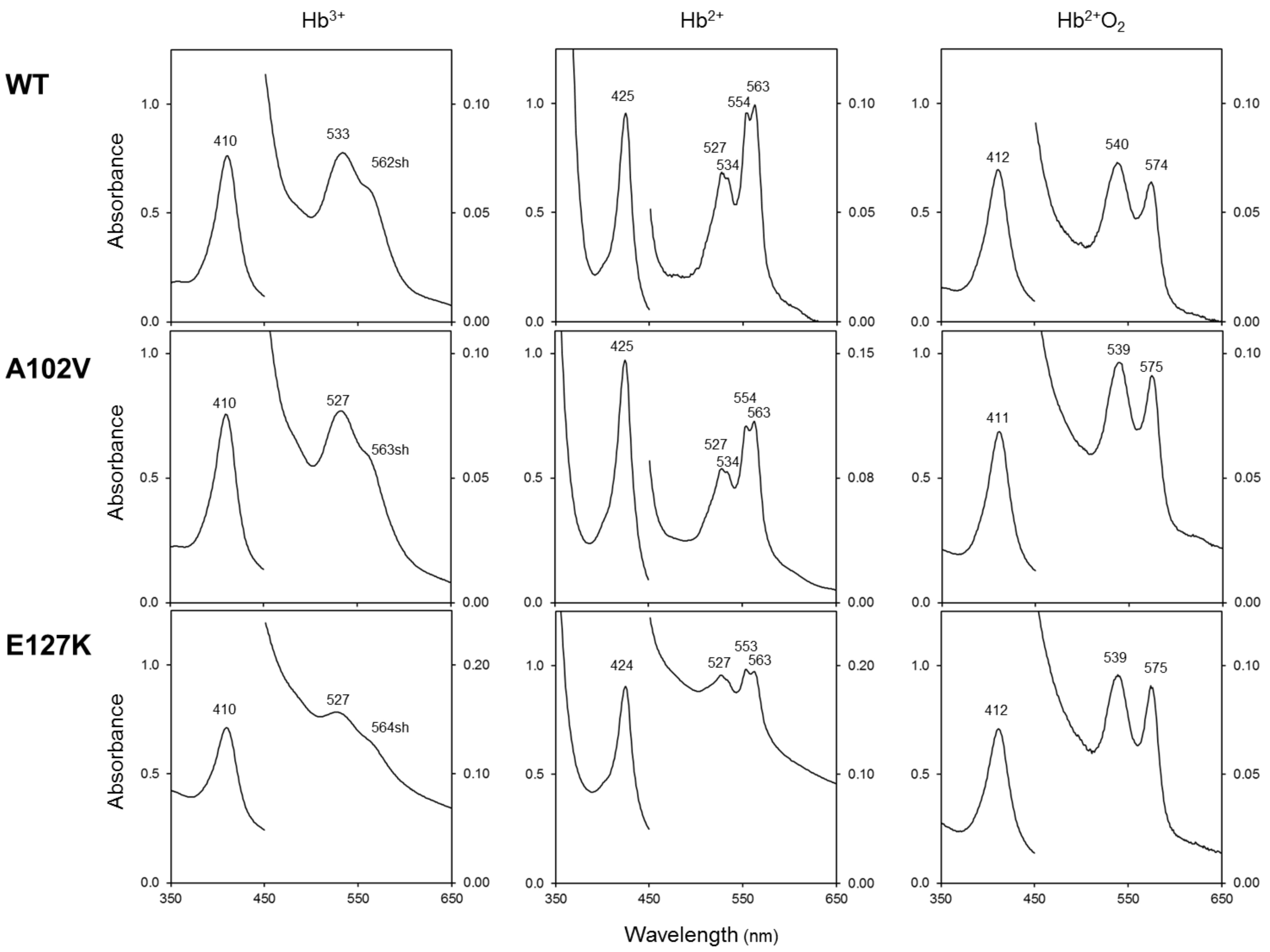

Figure S4: Representative Soret-visible spectra of recombinant WT, A102V, and E127K proteins. These were produced in *E. coli* and purified by ammonium sulfate fractionation and metal-affinity chromatography. The spectra of the ferric (Hb^{3+}), deoxyferrous (Hb^{2+}), and oxyferrous ($Hb^{2+}O_2$) proteins were obtained with 50 μM protein in 50 mM potassium phosphate buffer (pH 7.0). The spectra show relevant peaks and shoulders (sh) in nm.
